# Supplementary material for: Diffusion-Weighted MRI and Human Papillomavirus (HPV) Status in Oropharyngeal Cancer
Source: Cancers (Basel). 2024 Dec 23;16(24):4284. doi: 10.3390/cancers16244284 (PMC11674353; doi:10.3390/cancers16244284)
Supplement: Supplementary file 1 [file cancers-16-04284-s001.zip › cancers-3347485-supplementary updated.pdf]

Supplementary Table S1: Texture parameters pre-treatment according to HPV status

Texture parameter	HPV negative Median [IQR]	HPV positive Median [IQR]	P-value
original_shape_Elongation	0.68 [0.55;0.77]	0.64 [0.54;0.78]	0.84
original_shape_Flatness	0.29 [0.19;0.40]	0.29 [0.21;0.43]	0.65
original_shape_LeastAxis	4.65 [3.26;6.29]	4.46 [2.91;6.40]	0.69
original_shape_MajorAxis	16.35 [10.83;24.07]	14.57 [9.39;28.31]	0.50
original_shape_Maximum2DDiameterColumn	14.16 [8.06;20.88]	11.11 [8.15;22.11]	0.57
original_shape_Maximum2DDiameterRow	14.02 [8.25;21.21]	12.06 [8.06;23.05]	0.73
original_shape_Maximum2DDiameterSlice	15.58 [10.44;24.52]	14.49 [9.22;27.36]	0.66
original_shape_Maximum3DDiameter	18.53 [11.23;26.02]	15.96 [9.73;28.25]	0.48
original_shape_MinorAxis	10.34 [6.75;15.82]	8.95 [6.40;17.81]	0.64
original_shape_Sphericity	0.62 [0.51;0.70]	0.64 [0.55;0.76]	0.16
original_shape_SurfaceArea	466.62 [182.37;947.97]	356.89 [184.93;1174.70]	0.67
original_shape_SurfaceVolumeRatio	1.10 [0.82;1.38]	1.07 [0.80;1.36]	0.62
original_shape_Volume	439.00 [133.00;1168.00]	338.50 [161.50;1293.00]	0.88
original_firstorder_10Percentile	86.45 [72.00;105.00]	78.75 [67.50;90.00]	0.03
original_firstorder_90Percentile	149.00 [118.90;175.00]	142.00 [111.50;163.20]	0.21
original_firstorder_Energy	6.33E6 [1.81E6;2.23E7]	3.75E6 [1.32E6;1.66E7]	0.45
original_firstorder_Entropy	1.96 [1.64;2.34]	1.95 [1.52;2.37]	0.67
original_firstorder_InterquartileRange	29.12 [22.00;41.00]	30.12 [20.62;40.00]	0.68
original_firstorder_Kurtosis	3.12 [2.84;3.84]	3.16 [2.69;3.91]	0.45
original_firstorder_Maximum	196.50 [146.00;241.00]	169.00 [145.50;255.50]	0.41
original_firstorder_MeanAbsoluteDeviation	18.34 [14.41;25.04]	18.12 [12.69;24.75]	0.63
original_firstorder_Mean	119.88 [96.92;137.50]	107.47 [89.68;126.49]	0.08
original_firstorder_Median	116.00 [95.50;137.00]	105.00 [88.75;124.25]	0.06
original_firstorder_Minimum	48.50 [22.00;63.00]	39.00 [3.50;56.50]	0.22
original_firstorder_Range	139.50 [107.00;207.00]	144.50 [94.00;230.00]	0.56

original_firstorder_RobustMeanAbsoluteDeviation	12.19 [9.70;16.70]	13.14 [9.02;16.43]	0.75
original_firstorder_RobustMeanAbsoluteDeviation _1	122.02 [99.36;143.30]	110.53 [91.31;129.38]	0.10
original_firstorder_Skewness	0.054 [-0.37;0.35]	0.15 [-0.10;0.38]	0.23
original_firstorder_TotalEnergy	6.33E6 [1.81E6;2.23E7]	3.75E6 [1.32E6;1.66E7]	0.45
original_firstorder_Uniformity	0.30 [0.23;0.38]	0.30 [0.23;0.42]	0.79
original_firstorder_Variance	533.75 [334.86;982.88]	535.49 [260.10;1020.70]	0.57
original_glcm_Autocorrelation	13.74 [8.97;24.83]	12.68 [6.62;23.24]	0.20
original_glcm_ClusterProminence	21.46 [6.47;73.82]	13.06 [3.97;56.99]	0.29
original_glcm_ClusterShade	0.06 [0.46;1.78]	0.15 [-0.36;1.80]	0.78
original_glcm_ClusterTendency	2.46 [1.37;4.55]	2.01 [1.02;4.13]	0.29
original_glcm_Contrast	1.15 [0.85;2.03]	1.20 [0.765;2.418]	0.96
original_glcm_Correlation	0.30 [0.19;0.40]	0.18 [0.08;0.29]	<0.01
original_glcm_DifferenceAverage	0.75 [0.63;1.03]	0.80 [0.61;1.16]	0.91
original_glcm_DifferenceEntropy	1.49 [1.32;1.76]	1.49 [1.23;1.85]	0.89
original_glcm_DifferenceVariance	0.58 [0.42;0.89]	0.56 [0.40;1.02]	0.94
original_glcm_Id	0.69 [0.61;0.72]	0.66 [0.58;0.72]	0.82
original_glcm_Idm	0.67 [0.57;0.71]	0.64 [0.54;0.71]	0.86
original_glcm_Idmn	0.97 [0.97;0.98]	0.97 [0.96;0.98]	0.08
original_glcm_Idn	0.90 [0.89;0.92]	0.89 [0.88;0.91]	0.17
original_glcm_Imc1	-0.11 [-0.14;-0.08]	-0.10 [-0.14;-0.07]	0.28
original_glcm_Imc2	0.51 [0.44;0.64]	0.50 [0.39;0.59]	0.31
original_glcm_InverseVariance	0.46 [0.44;0.48]	0.46 [0.43;0.49]	0.39
original_glcm_JointAverage	3.67 [2.99;4.90]	3.54 [2.57;4.80]	0.20
original_glcm_JointEnergy	0.12 [0.07;0.17]	0.12 [0.07;0.20]	0.73
original_glcm_JointEntropy	3.48 [3.02;4.34]	3.51 [2.72;4.35]	0.60
original_glcm_MaximumProbability	0.23 [0.14;0.29]	0.22 [0.14;0.31]	0.65
original_glcm_SumAverage	7.38 [5.99;9.72]	7.07 [5.14;9.60]	0.20
original_glcm_SumEntropy	2.50 [2.14;2.98]	2.40 [1.95;2.94]	0.36
original_glcm_SumSquares	0.87 [0.57;1.61]	0.82 [0.44;1.64]	0.46
original_gldm_DependenceEntropy	5.49 [4.91;5.93]	5.32 [4.78;5.78]	0.36

original_gldm_DependenceNonUniformity	31.90 [11.78;75.65]	30.40 [12.17;90.72]	0.78
original_gldm_DependenceNonUniformityNormalized	0.08 [0.06;0.10]	0.079 [0.06;0.10]	0.95
original_gldm_DependenceVariance	15.01 [9.32;22.53]	14.88 [9.65;25.37]	0.70
original_gldm_GrayLevelNonUniformity	114.68 [49.49;317.45]	111.63 [41.317;493.79]	0.97
original_gldm_GrayLevelVariance	0.96 [0.59;1.67]	0.94 [0.50;1.71]	0.57
original_gldm_HighGrayLevelEmphasis	14.73 [9.68;26.38]	13.37 [7.54;25.34]	0.27
original_gldm_LargeDependenceEmphasis	73.04 [43.30;115.57]	70.05 [42.44;118.09]	0.84
original_gldm_LargeDependenceHighGrayLevelEmphasis	985.84 [626.25;1736.60]	933.40 [413.34;1336.50]	0.36
original_gldm_LargeDependenceLowGrayLevelEmphasis	6.59 [3.06;11.29]	7.66 [3.01;15.79]	0.32
original_gldm_LowGrayLevelEmphasis	0.10 [0.05;0.16]	0.11 [0.06;0.24]	0.16
original_gldm_SmallDependenceEmphasis	0.07 [0.05;0.11]	0.07 [0.04;0.12]	0.88
original_gldm_SmallDependenceHighGrayLevelEmphasis	1.44 [0.71;2.48]	1.32 [0.60;2.85]	0.66
original_gldm_SmallDependenceLowGrayLevelEmphasis	0.01 [0.00;0.02]	0.01 [0.01;0.02]	0.42
original_glrlm_GrayLevelNonUniformity	79.00 [36.22;197.18]	69.51 [32.38;225.24]	0.93
original_glrlm_GrayLevelNonUniformityNormalized	0.28 [0.22;0.34]	0.28 [0.21;0.37]	0.86
original_glrlm_GrayLevelVariance	1.11 [0.70;1.89]	1.06 [0.62;1.89]	0.57
original_glrlm_HighGrayLevelRunEmphasis	15.39 [9.89;27.12]	13.55 [7.87;25.67]	0.26
original_glrlm_LongRunEmphasis	2.49 [1.94;3.44]	2.55 [1.89;3.71]	0.78
original_glrlm_LongRunHighGrayLevelEmphasis	44.66 [22.62;66.43]	37.26 [19.46;62.39]	0.41
original_glrlm_LongRunLowGrayLevelEmphasis	0.26 [0.134;0.426]	0.32 [0.15;0.59]	0.21
original_glrlm_LowGrayLevelRunEmphasis	0.10 [0.06;0.17]	0.12 [0.06;0.24]	0.16
original_glrlm_RunEntropy	3.10 [2.70;3.44]	3.06 [2.69;3.39]	0.67
original_glrlm_RunLengthNonUniformity	191.58 [64.66;473.86]	168.71 [54.58;552.17]	0.68
original_glrlm_RunLengthNonUniformityNormalized	0.61 [0.52;0.68]	0.62 [0.540;0.69]	0.84
original_glrlm_RunPercentage	0.74 [0.67;0.81]	0.76 [0.67;0.81]	0.93
original_glrlm_RunVariance	0.56 [0.35;0.92]	0.61 [0.33;1.04]	0.82
original_glrlm_ShortRunEmphasis	0.80 [0.74;0.85]	0.80 [0.75;0.85]	0.87
original_glrlm_ShortRunHighGrayLevelEmphasis	12.74 [7.89;22.61]	11.04 [6.51;22.73]	0.27
original_glrlm_ShortRunLowGrayLevelEmphasis	0.08 [0.05;0.13]	0.09 [0.05;0.17]	0.17

original_glszm_GrayLevelNonUniformity	6.11 [3.00;11.02]	5.79 [2.58;11.32]	0.97
original_glszm_GrayLevelNonUniformityNormalized	0.20 [0.17;0.27]	0.22 [0.16;0.29]	0.40
original_glszm_GrayLevelVariance	2.76 [1.84;4.48]	2.59 [1.58;5.06]	0.74
original_glszm_HighGrayLevelZoneEmphasis	18.33 [11.06;33.70]	17.96 [10.84;31.27]	0.62
original_glszm_LargeAreaEmphasis	1451.10 [391.91;5647.60]	1371.30 [205.88;11434.00]	0.89
original_glszm_LargeAreaHighGrayLevelEmphasis	19052.00 [6151.60;89419.00]	17626.00 [2959.90;130887.00]	0.87
original_glszm_LargeAreaLowGrayLevelEmphasis	136.07 [30.30;485.83]	127.62 [31.64;937.73]	0.70
original_glszm_LowGrayLevelZoneEmphasis	0.16 [0.08;0.27]	0.17 [0.11;0.30]	0.26
original_glszm_SizeZoneNonUniformity	6.23 [3.50;17.63]	6.96 [3.00;15.36]	0.88
original_glszm_SizeZoneNonUniformityNormalized	0.24 [0.20;0.32]	0.27 [0.22;0.33]	0.37
original_glszm_SmallAreaEmphasis	0.49 [0.40;0.57]	0.49 [0.43;0.56]	0.57
original_glszm_SmallAreaHighGrayLevelEmphasis	9.18 [5.50;17.29]	10.16 [5.10;17.83]	0.98
original_glszm_SmallAreaLowGrayLevelEmphasis	0.07 [0.04;0.13]	0.07 [0.05;0.14]	0.53
original_glszm_ZoneEntropy	3.99 [3.02;4.77]	3.74 [2.87;4.73]	0.55
original_glszm_ZonePercentage	0.06 [0.04;0.12]	0.06 [0.04;0.13]	0.90
original_glszm_ZoneVariance	1045.40 [293.72;5390.40]	1158.80 [139.10;10314.00]	0.90
original_ngtdm_Busyness	2.383 [1.22;4.93]	2.55 [1.04;6.72]	0.46
original_ngtdm_Coarseness	0.02 [0.01;0.04]	0.02 [0.01;0.04]	0.95
original_ngtdm_Complexity	10.14 [5.12;23.79]	9.63 [4.75;30.67]	0.67
original_ngtdm_Contrast	0.03 [0.02;0.05]	0.04 [0.03;0.05]	0.47
original_ngtdm_Strength	0.30 [0.13;0.53]	0.23 [0.09;0.47]	0.35

IQR=[Q1; Q3], Q1: first quartile, Q3: third quartile

P-value from Mann-Whitney U test

Supplementary Table S2: Association pre-treatment ADC values and outcome

		LRC	
**Predictor**	**Test**	**HR (95%CI)**	**P-value**
MRI 1_original_firstorder_10Percentile	x2 units	2.276 (1.115;4.647)	0.0239
MRI 1_original_firstorder_Mean	x2 units	1.853 (1.029;3.338)	0.0399
MRI 1_original_firstorder_Median	x2 units	1.771 (0.998;3.143)	0.0508
MRI1_original_firstorder_90Percentile	x2 units	1.755 (1.019;3.023)	0.0425
MRI1_original_firstorder_Maximum	x2 units	1.811 (1.082;3.032)	0.0239

Predictors were log-transformed and hazard ratios are then estimated for a two-fold increase (doubling) of the predictor variable. Hazard ratios are then estimated for a two-fold increase (doubling) of the predictor variable. HR: hazard ratio, CI: confidence interval HR>(<)1:higher (lower) event risk for ADC level. P-values from Cox model

Supplementary Table S3: Association mid-treatment ADC values (week 4) and Δ ADC and outcome

Predictor	Test	LRC HR (95%CI)	LRC P-value
MRI 2_original_firstorder_10Percentile	x2 units	0.41 (0.25;0.65)	<0.01
MRI 2_original_firstorder_Mean	x2 units	0.39 (0.22;0.69)	<0.01
MRI 2_original_firstorder_Median	x2 units	0.39 (0.22;0.70)	<0.01
MRI2_original_firstorder_90Percentile	x2 units	0.40 (0.21;0.74)	<0.01
MRI2_original_firstorder_Maximum	x2 units	0.43 (0.22;0.82)	0.01

Predictors were log-transformed and hazard ratios are then estimated for a two-fold increase (doubling) of the predictor variable. HR: hazard ratio, CI: confidence interval HR>(<)1:higher (lower) event risk for ADC level. P-values from Cox model

Predictor	Test	LRC HR (95%CI)	LRC P-value
Delta_mean_ADC (Δ ADC)	+10 units	0.89 (0.84;0.94)	<0.01

Linear effect was modelled and the hazard ratio is estimated for a 10-unit increase of the predictor variable. HR: hazard ratio, CI: confidence interval HR>(<)1:higher (lower) event risk for ADC level. P-values from Cox model
